# Novel Use of an Electroceutical Wound Dressing to Complement Wound Closure in High-Risk Sternotomy Patients: Clinical Safety and Outcomes

**DOI:** 10.7759/cureus.78405

**Published:** 2025-02-03

**Authors:** Zain Khalpey, Zacharya I Khalpey, Tyler Phillips, Pranav Jutla, Ujjawal Kumar, Feras H Khaliel

**Affiliations:** 1 Department of Cardiothoracic Surgery, HonorHealth, Scottsdale, USA; 2 Khalpey AI Lab, Applied & Translational AI Research Institute (ATARI), Scottsdale, USA; 3 School of Life Sciences, Arizona State University, Tempe, USA; 4 School of Clinical Medicine, University of Cambridge, Cambridge, GBR; 5 Department of Cardiac Surgery, King Faisal Specialist Hospital and Research Centre, Riyadh, SAU

**Keywords:** cardiovascular surgery, electroceutical wound dressing, frailty, obesity, sternotomy

## Abstract

Objectives

Surgical wound separation, known as wound dehiscence, occurs when the layers of a surgical incision pull apart either partially or completely. This condition, alongside wound infections, presents a major challenge in surgical practice. The consequences can be severe, ranging from compromised healing and increased susceptibility to infection to higher medical expenses and significant patient distress. Particularly in cardiac surgery involving median sternotomy, patients with elevated risk factors require careful attention to wound closure methods. Recent advances in medical technology have introduced innovative solutions for post-sternotomy wound management. A promising development in this field is the emergence of electroceutical wound dressings (EWDs). EWDs are one such example. EWDs replicate the physiological electrical signal generated during the time of injury, thus serving a dual purpose: enhancing the healing process by enabling cell proliferation toward the site of injury while also serving as a robust antimicrobial device to prevent wound infections.

Methods

This study examined a cohort of 100 patients undergoing cardiac surgery via median sternotomy at a single institution by a single surgeon. The sternotomy wound was closed in the usual fashion and covered with an EWD. Demographics, medical histories, and the occurrence of sternal complications were collected for each patient, followed by the statistical evaluation of collected data.

Results

At their 14- and 30-day follow-up visits, none of the patients had experienced sternal wound dehiscence or infection, and their sternotomy wounds showed excellent signs of normal wound closure. A comprehensive sternal pain evaluation was carried out, and no significant pain was elicited in any patients, a sign that sternal closure was successful and stable. The addition of the EWD to our clinical practice has also contributed to no longer requiring postoperative chest stabilization adjuncts, resulting in significant financial and resource savings for our group.

Conclusions

This study showed the promise of the EWD as an effective solution to stabilize sternal wound closure in high-risk patients. Its biomimetic and robust antimicrobial properties directly address the specific challenges faced by these high-risk individuals. The EWD offers an unprecedented and modern approach to wound closure in populations vulnerable to complications.

## Introduction

Even with the significant progress that has been made with minimally invasive techniques, a median sternotomy remains the most dependable approach, considering the unrivaled exposure of the heart and great vessels [1]. However, this procedure is not without substantial risks, particularly for patients with high-risk profiles. Severe complications of sternal dehiscence and deep sternal wound infections (DSWIs) consist of prolonged recovery times, extended hospital stays, higher healthcare resource utilization, and poor prognoses. Alarmingly, these sternal complications are responsible for nearly 20% of hospital readmissions within 30 days [2], highlighting their profound impact on both patient outcomes and healthcare costs while stressing the importance of addressing this issue.

Current preventative strategies, including prophylactic antibiotics, antibiotic-infused surgical sites, advanced techniques for sternal closure, and negative pressure dressings, have helped reduce but not eliminate these risks. High-risk groups, such as patients with obesity, diabetes, frailty, immunosuppression, or those undergoing repeat surgeries, continue to experience sternal complication rates of up to 10% [3]. Even with the implementation of optimal care, these patients still face a persistent complication rate of 3%-5%. This has led us to consider and advocate for additional interventions aimed at improving postoperative surgical outcomes and reducing complications for vulnerable populations.

The use of bioelectric and electroceutical wound dressings (EWDs) is gaining attention as a novel approach to combating biofilm-associated infections and promoting wound healing, particularly in chronic and hard-to-treat wounds [4,5]. For instance, one study demonstrated that EWDs, when activated by conductive wound exudate to generate a low electric field, disrupt the biofilm structure of *Pseudomonas aeruginosa*, a common pathogen in chronic wound infections [4]. Crucially, this study showed that silver dressings alone, which previously were used for their antimicrobial properties, were unable to disrupt the biofilm. This dressing showed a significant reduction in biofilm thickness and a decrease in live bacterial cells, alongside repressed expression of quorum-sensing genes and a reduction in biofilm integrity, which silver dressings failed to achieve. The EWD also produces reactive oxygen species (ROS), which contribute to its bactericidal effects, providing a unique mechanism that enhances its antibiofilm properties, especially against resistant strains.

Additionally, bioelectric dressings have shown promise in accelerating wound healing, particularly in skin graft donor sites. In a study involving patients who underwent skin grafting, the application of bioelectric dressing alongside standard care led to a 34.62% faster epithelialization compared to control groups [6]. Patients also reported improved scar outcomes in terms of color, stiffness, and overall quality, suggesting the dressings’ potential to enhance healing and patient satisfaction. This dressing’s efficacy extends to reducing bacterial colonization, as seen in shoulder surgeries where it significantly lowered the skin burden of *Cutibacterium acnes*, potentially reducing perioperative infection risks [7]. Collectively, these studies underscore the potential of bioelectric and electroceutical dressings as effective, nonpharmacological interventions in wound care, with benefits spanning accelerated healing and reduced infection risk.

Our study investigated the potential of an EWD (JumpStart, Arthrex Inc., Naples, FL), in enhancing sternal wound closure in cardiac surgery patients at high risk of postoperative sternal complications. We suggest that using an EWD as part of an enhanced wound closure strategy will reduce sternal complications in such patients. We aim to assess the efficacy of this model approach by evaluating key outcomes such as length of admission to the intensive care unit and the hospital, the incidence of postoperative sternal wound infections and sternal wound dehiscence, as well as postoperative pain at follow-up. Using these outcomes, we aim to assess whether EWDs may help address the current issue of sternal wound complications in cardiac surgery.

## Materials and methods

This retrospective observational study included patients undergoing cardiac surgery via median sternotomy at our institution, HonorHealth, Scottsdale, AZ, USA. An Institutional Review Board (IRB) approval was granted (IRB-23-0025, March 30, 2023), and informed consent was obtained for every single patient about the relevant surgical procedures as well as an anonymized inclusion into this study. All study methodologies adhered to the proper regulations for working with human subjects and the updated Declaration of Helsinki [8]. Patients with previous sternal wound complications and those under 18 years of age were excluded.

Notably, the sternal wound complications (infections and dehiscence) were defined using the Society of Thoracic Surgeons (STS) criteria [9]. Heart failure was defined using the American Heart Association (AHA) criteria for diastolic heart failure [10]. With regard to chronic kidney disease (CKD), we defined it using an internationally conventional set of guidelines from the Kidney Disease: Improving Global Outcomes (KDIGO) [11].

Data collection

Data about a patient’s demographics, preoperative clinical characteristics, operative characteristics, and postoperative outcomes were collected [12], anonymized, and then securely stored as per the standard, institutional protocols for outcomes research data. Furthermore, the preoperative data collected included key surgical risk scores (STS mortality and DSWI risk scores). Along with that, data regarding risk factors for sternal complications such as prior sternotomy, chronic obstructive pulmonary disease (COPD), a positive smoking history, or long-term immunosuppressive medication use, e.g., steroids were also noted. Data were also collected for other comorbidities and other key risk scores.

Operative technique and clinical protocol

**Figure 1:**
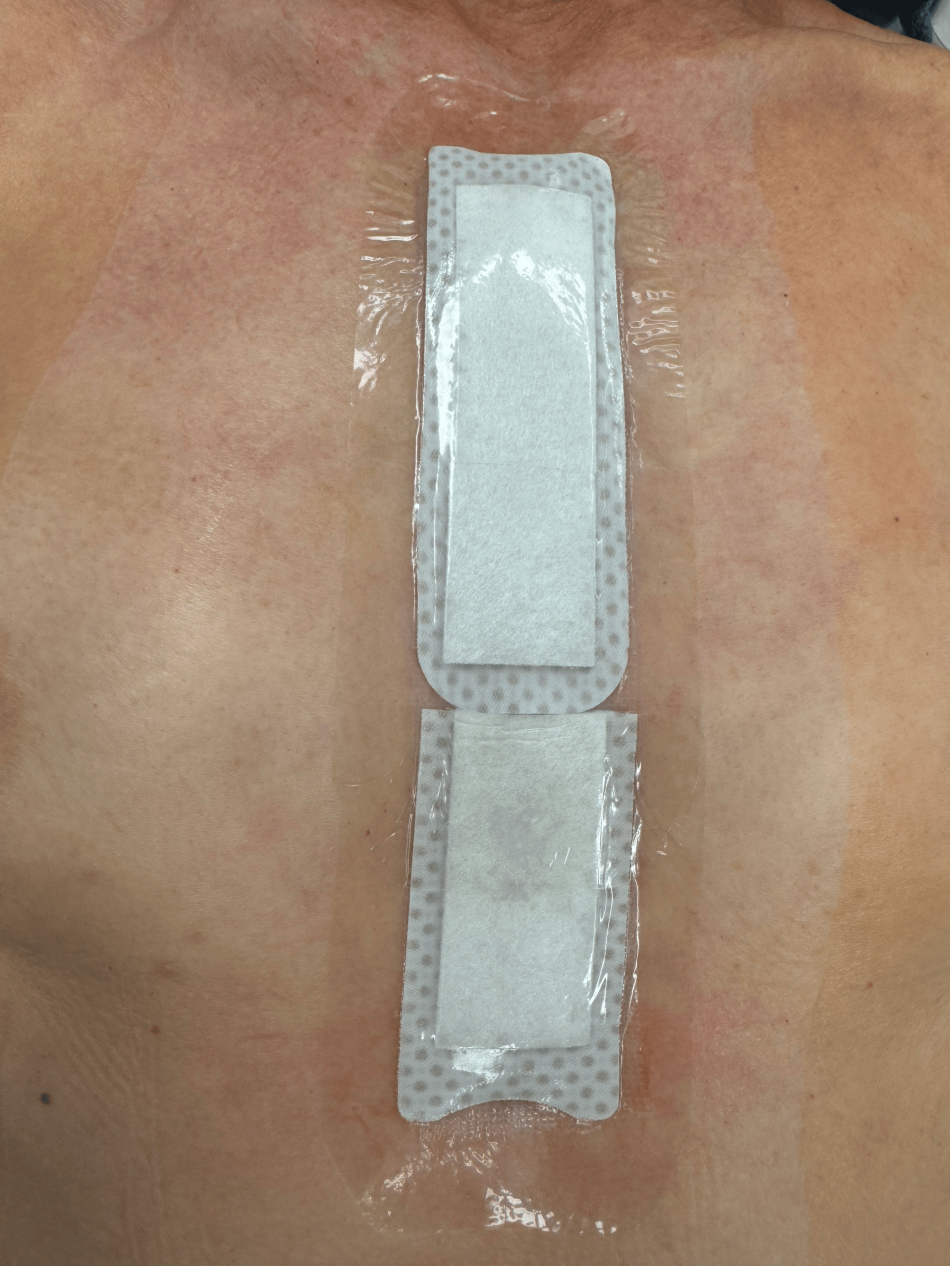
A sternotomy wound that has been closed in the usual fashion and covered with a JumpStart electroceutical wound dressing

In all patients, sternal closure was achieved using our enhanced healing protocol. This includes sternal closure using ultrahigh molecular weight polyethylene suture tapes (TigerTape and FiberTape, Arthrex Inc., Naples, FL) rather than steel wires [13]. The suture tapes are presoaked in vancomycin solution for five minutes before use. In figure-of-eight patterns, four sutures are placed through the manubrium and sternal interspaces around the sternum. Each figure-of-eight pattern of suture tape is sequentially tightened, applying between 60 and 80 lb of pressure using the Arthrex tensioner, and a half-hitch knot is tied to lock the sutures. Next, the wound is washed with a vancomycin solution, and 160 mg of aseptically processed amnion-chorion placental allograft (aACPA) (Salera®, MTF Biologics, Edison, NJ) are added to the sternum and subcutaneous tissues before closure [14,15], which are closed sequentially with 0, 2-0, and 4-0 Stratafix suture (Ethicon Inc., Cincinnati, OH). The wound is dressed with a JumpStart FlexEFit EWD (Figure 1, Arthrex Inc.).

Follow-up and outcomes

Postoperative data collection included the total duration of stay in the ICU and hospital (length of stay (LOS)) as well as critical outcomes such as hospital mortality, sternal wound infections, and dehiscence. In line with standard protocols, cardiac surgery patients underwent follow-up evaluations at two weeks and one month post-surgery. These follow-ups involved comprehensive assessments of multiple physiological systems and a review of prescribed medications. Pain was classified as significant if it substantially interfered with the patient’s daily activities or recovery process, or if a previously unprescribed opioid was newly initiated. Each follow-up involved a detailed wound examination and pain assessment. During the evaluation, the surgeon applied pressure to the breastbone and intercostal spaces to gauge pain levels. Additionally, while the patient was standing, the surgeon placed two fingers on the breastbone and instructed the patient to rotate their upper body laterally to assess pain responses.

Statistical analyses

The continuous variables of mean and standard deviation (SD) were presented for normally distributed data, while nonparametric data were presented as a median with an interquartile range. Categorical variables were presented in the format N (%). All statistical analyses and visualizations were undertaken using R v4.4.2 (R Foundation, Vienna, Austria) [16].

## Results

Patient characteristics

**Figure 2:**
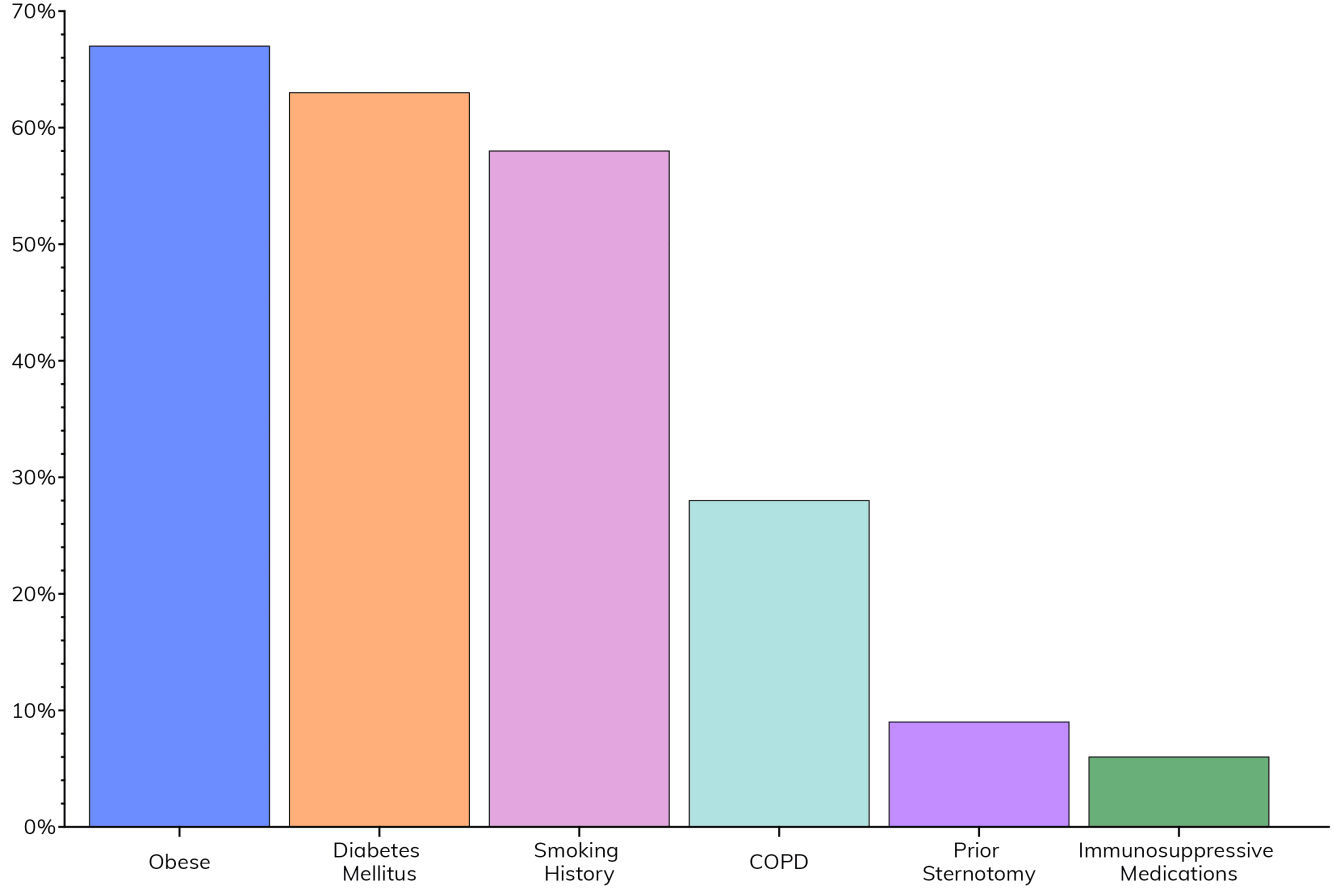
Prevalence of risk factors for sternal complications within the study population COPD: chronic obstructive pulmonary disease

This study included 100 patients with various risk factors for postoperative sternal complications (Table 1). As seen in Figure 2, obesity was the most prevalent, with a mean population BMI of 33.48 ± 1.11 kg/m^2^. Diabetes mellitus and a smoking history were also common. Less common risk factors included the use of immunosuppressive medications, prior sternotomy, and COPD.

**Table 1:** Relevant risk factors for sternal complications Categorical variables are represented as N (%) with parametric continuous variables being represented as mean ± SD and nonparametric continuous variables represented as median (LQ-UQ)

Variable	Value
Number	100
Body mass index (kg/m^2^)	33.48 ± 1.11
Obese	67 (67%)
Diabetes mellitus	63 (63%)
Smoking history	58 (58%)
Prior sternotomy	9 (9%)
Chronic obstructive pulmonary disease	28 (28%)
Immunosuppressive medications	6 (6%)

Considering other preoperative characteristics (Table 2), the mean patient age was 65 years, with a male predominance. Hypertension was common, as were hyperlipidemia and coronary artery disease. The median STS score was 1.77, and the median STS DSWI score was 0.241. Other notable comorbidities included obstructive sleep apnea, heart failure, and CKD. The median CHA2DS2-VASc and HAS-BLED scores were 2 and 1, indicating moderate risk profiles for thromboembolic and bleeding complications.

**Table 2:** Other preoperative characteristics and comorbidities STS: Society of Thoracic Surgeons; DSWI: deep sternal wound infection; CHA2DS2-VASc: risk score for stroke risk for patients with atrial fibrillation; HAS-BLED: risk score for major bleeding for anticoagulated patients Categorical variables are represented as N (%) with parametric continuous variables being represented as mean ± SD and nonparametric continuous variables represented as median (LQ-UQ)

Variable	Value
Number	100
Age (y)	65 ± 2
Sex: male	60 (60%)
STS score	1.77 (1.19 - 2.5)
STS DSWI score	0.241 (0.124 - 0.258)
Hypertension	86 (86%)
Hyperlipidemia	62 (62%)
Coronary artery disease	62 (62%)
Obstructive sleep apnea	36 (36%)
Heart failure	26 (26%)
Chronic kidney disease	11 (11%)
Prior Atrial Fibrillation	25 (25%)
Prior percutaneous coronary intervention	16 (16%)
Prior myocardial infarction	18 (18%)
Prior stroke	9 (9%)
CHA2DS2-VASc	2 (1 - 3)
HAS-BLED	1 (1 - 2)

Operative characteristics

**Figure 3:**
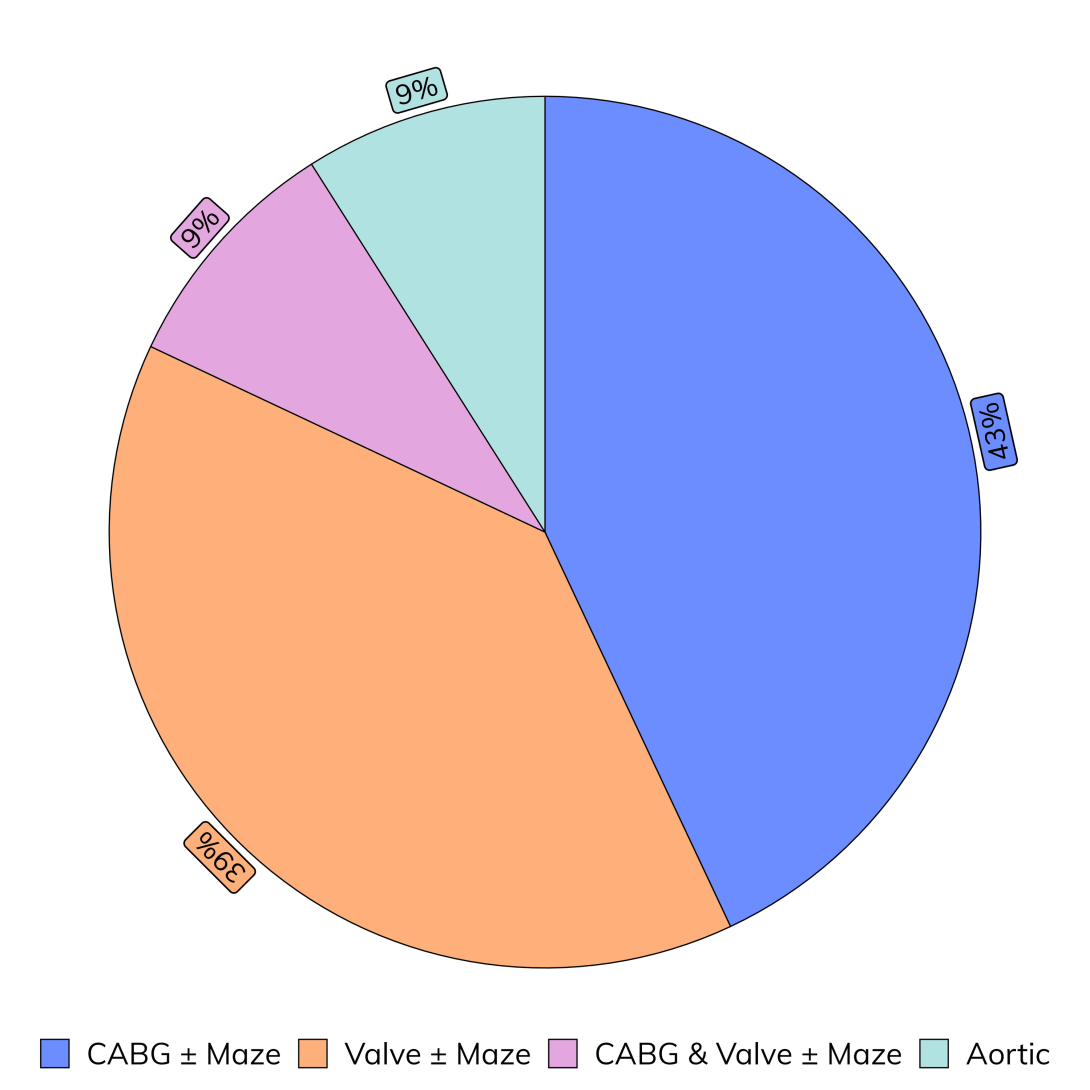
Case mix of patients that underwent sternal closure with the addition of EWD EWD: electroceutical wound dressing; CABG: coronary artery bypass graft

Table 3 highlights the case mix and the operative characteristics of patients who underwent sternal closure with the addition of EWD, shown in Figure 3. The mean operative time was 243 minutes, the mean cardiopulmonary bypass time was 83 minutes, and the mean aortic cross-clamp time was 64 minutes. These results show that the EWD was used without substantially prolonging operative times.

**Table 3:** Operative characteristics CABG: coronary artery bypass graft Categorical variables are represented as N (%) with parametric continuous variables being represented as mean ± SD and nonparametric continuous variables represented as median (LQ-UQ)

Variable	Value
Elective	82 (82%)
Procedure type	
CABG ± maze	43 (43%)
Valve ± maze	39 (39%)
CABG & valve ± maze	9 (9%)
Aortic	9 (9%)
Cardiopulmonary bypass (min)	83 ± 4
Aortic cross-clamp (min)	64 ± 3
Operative time (min)	243 ± 16

Patient outcomes

**Figure 4:**
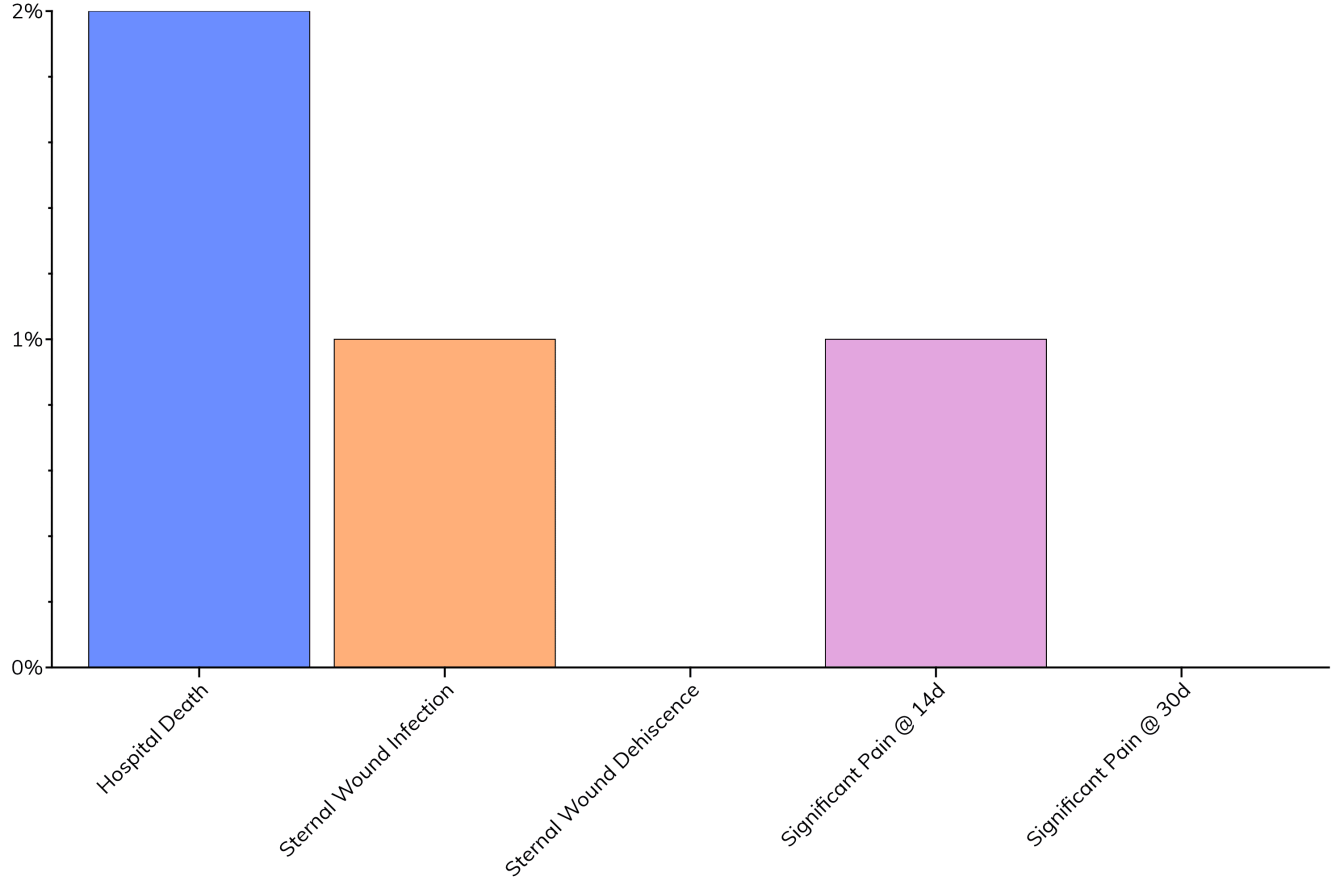
Postoperative outcomes Categorical variables are represented as N (%) with parametric continuous variables being represented as mean ± SD and nonparametric continuous variables represented as median (LQ-UQ)

Postoperative outcomes are shown in Table 4 and Figure 4. The median ICU admission duration was three days, while the overall hospital admission rested at a median duration of six days. Among the cohort, no in-hospital deaths were reported. Following cardiac surgery, significant postoperative complications, such as sternal wound infections or dehiscence, did not occur in this complex patient population, which notably had at least one sternal risk factor. The absence of sternal complications suggests that EWD may support wound closure and stability. Additionally, there were no reports of significant pain during either the 14-day or 30-day postoperative follow-up appointments. The lack of substantial pain suggests that patient comfort was well-managed. This lack of persistent pain could potentially be attributed to the stability provided by the closure technique, with better wound healing.

**Table 4:** Postoperative outcomes LOS: length of stay Categorical variables are represented as N (%) with parametric continuous variables being represented as mean ± SD and nonparametric continuous variables represented as median (LQ-UQ)

Variable	Value
ICU LOS	3 (2-7)
Hospital LOS	7 (5-9)
Hospital death	2 (2%)
Sternal wound infection	1 (1%)
Sternal wound dehiscence	0 (0%)
Significant pain @ 14d	1 (1%)
Significant pain @ 30d	0 (1%)

Together, the outcomes indicate that the use of EWD for covering sternotomy wounds might be associated with an uncomplicated recovery; this is noted by the absence of major sternal complications and the presence of effective pain control. Hospital and ICU stays are within the reasonable ranges for this sort of cardiac surgical case mix, which underscores the potential benefits of this closure method in facilitating patient recovery, and even potentially enabling timely discharge.

Financial comparison

The implementation of the JumpStart EWD into our enhanced wound closure protocol has significant potential to reduce financial and healthcare resource costs. For wire closure with necessary adjuncts in high-risk patients, costs ranged from $1,055 to $1,385 per patient. This cost includes costs of between $80 and $160 for the sternal wires (eight per patient, $10 to $20 each [17]), $225 for a support vest [18], and $750 to $1,000 for the Prevena system (three days of use: therapy unit and dressing costing between $500 and $600, the dressing kit costing between $200 and $300, and the canister for fluid collection costing between $50 and $100) [19]. In contrast, closure with our enhanced closure formula costs $450 per obese patient (four suture tapes, $100 each [20], and two EWD strips, $25 each [21]). This change in our wound closure strategy resulted in savings of $605 to $935 per obese patient compared to wire closure. This implies that the use of new technologies such as this EWD with the suture tape system confers significant cost savings, most significantly by eliminating the use of support devices in obese patients.

## Discussion

The use of EWDs in high-risk cardiac surgery patients undergoing median sternotomy has shown promising results. Our results imply that EWDs may support wound healing and therefore reduce complications in a patient population at high risk of sternal wound issues.

Key findings and implications

Our study population consisted of 100 patients. All patients had at least one risk factor for sternal wound complications. Notably, 67% were obese, with a mean BMI of 33.48 kg/m^2^. Other common risk factors included hypertension and diabetes mellitus, present in 86% and 63% of the patients, respectively. Despite this high-risk profile, only 1% of cases progressed into sternal wound infections, and 1% of cases experienced significant pain at the 14-day assessment, reducing to 0% of cases expressing pain at the 30-day assessment. This outcome is positive given the typically raised complication rates in such patients. The absence of persistent pain could be connected to the enhanced wound stability provided by EWD use. The operative times suggest that using the EWD as part of the surgical protocol did not lead to significantly increased procedural times. That there were no in-hospital deaths in this high-risk group further highlights the benefits of the EWD’s use in supporting wound closure. Our analysis additionally demonstrates significant cost savings with the EWD as part of an enhanced wound closure protocol by eliminating the need for support/healing adjuncts in high-risk patients.

EWDs: how do they work and their potential in high-risk patients

**Figure 5:**
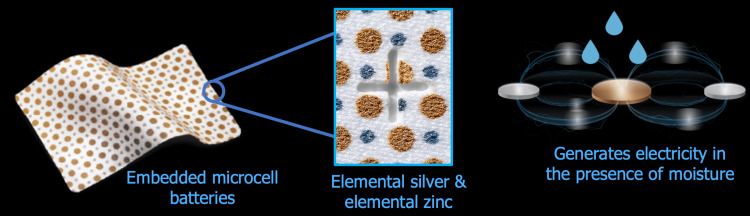
Mechanism of action of the JumpStart dressing Image adapted/reproduced from Arthrex Inc., with permission

EWDs, like JumpStart, are highly effective in promoting wound healing due to their ability to mimic yet enhance existing physiological mechanisms. These dressings generate a physiological level of electrical microcurrents (Figure 5) when activated by wound exudate [22], which are generated naturally by the body during times of injury. These microcurrents serve a dual purpose: they promote reepithelization and promote healing, and through the incorporation of the silver and zinc nanoparticles within the dressings, EWDs can kill bacteria by disrupting biofilm [23] and blocking the energy metabolism operating through bacterial membranes [22].

Studies have demonstrated the capacity of silver-zinc EWDs to disrupt biofilms formed by pathogens such as *Cutibacterium acnes* [7] and *Pseudomonas aeruginosa *[4], which are commonly associated with chronic and postoperative wound infections. However, biofilm integrity and bacterial colonization are severely inhibited using EWDs. For instance, microcurrent-generating dressings have significantly reduced *Cutibacterium acnes *colonization in patients who went through shoulder arthroplasty or arthroscopic shoulder surgery [7]. Furthermore, Banerjee et al. proved that the growth of the pathogenic* Pseudomonas ​​​​​aeruginosa *strain was significantly arrested using electroceutical dressing. They also showed that quorum sensing genes lasR and rhIR and the enzyme glycerol 3-phosphate dehydrogenase were also suppressed through the presence of microcurrents. Furthermore, these dressings induce the production of small amounts of ROS, enhancing their antimicrobial efficacy without damaging surrounding tissue [4]. By targeting bacterial colonization and impairing bacterial stability, electroceutical dressings create a more favorable environment for tissue repair while supporting infection prevention in vulnerable patients.

For especially high-risk patients, such as obese, diabetic, or immunocompromised individuals, the impaired wound healing process is often linked to chronic inflammation [24], resulting in worse health outcomes and potentially even the emergence of resistant microbial biofilms. By restoring the disrupted bioelectric field and providing enhanced antimicrobial protection, EWDs create a supportive environment for effective wound closure by supplementing crucial immune functions for high-risk and compromised individuals.

Comparison with previous research

The findings of this study align with and build upon prior research demonstrating the efficacy of electroceutical dressings in wound management. Prior research has shown that EWDs significantly accelerate wound closure and reduce bacterial colonization in diverse clinical settings [22]. For instance, previous studies have shown that microcurrent dressings reduced *Cutibaceterium acnes* skin colonization in shoulder arthroplasty patients, highlighting their potential to minimize both peri- and postoperative infection risks [7]. Additionally, Banerjee et al. demonstrated that silver-zinc electroceutical dressings disrupted biofilm formation and reduced bacterial viability by repressing quorum-sensing pathways and other key enzymes, effects that were not replicated with conventional silver-based dressings [4]. These findings underscore the unique advantage of bioelectric dressings in addressing resistant biofilms, a critical factor in managing wounds and postoperative infections.

This study extends these findings by demonstrating the efficacy of EWDs in high-risk cardiac surgery patients undergoing median sternotomy. Unlike previous studies that focused on skin graft sites, this research highlights the utility of EWDs in preventing DSWIs and dehiscence, two of the most challenging complications in cardiac surgery. The absence of any sternal wound complications in this study, despite the presence of significant risk factors, validates the efficacy and potential of EWDs to improve outcomes in high-risk populations.

Comparison with silver dressings

EWDs offer notable advantages over traditional silver dressings, particularly in managing complex wounds prone to infection and delayed healing. While silver dressings have been a mainstream method in wound care due to their antimicrobial properties, EWDs provide enhanced benefits in disrupting biofilms and accelerating healing. Silver dressings and EWDs both aim to enhance wound healing, but they operate through distinct mechanisms and exhibit varying degrees of effectiveness.

Silver dressings release silver ions upon contact with wound exudate. These ions possess broad-spectrum antimicrobial properties, disrupting bacterial cell walls, interfering with protein function, and inhibiting key biomolecular events and bacterial function. Thus, they reduce the microbial load in the wound [25]. By controlling infection, silver dressings help create a more conducive environment for the natural healing process.

EWDs, on the other hand, contain silver and zinc electrodes that generate electric fields, mimicking the body’s natural bioelectric signals involved in wound healing. These electrical stimulations can enhance cellular activities such as migration, proliferation, and collagen synthesis. Additionally, the electric fields can more efficiently disrupt bacterial biofilms thereby promoting a healthier wound environment [26].

Silver dressings release silver ions that exhibit antimicrobial activity by disrupting bacterial cell wall function [25]; however, their efficacy against established biofilms was proven to be limited. EWDs, on the other hand, generate electric fields that disrupt biofilm integrity more effectively. Evidently, Banerjee et al. demonstrated that an electroceutical dressing markedly disrupted *Psuedomonas aeruginosa *biofilms, whereas silver-only control dressings showed no significant decrease in biofilm integrity, cell viability, or extracellular polymeric substance formation [4].

EWDs not only combat infection but also promote tissue repair by mimicking the body’s natural electrical signals involved in wound healing. This dual action can lead to faster reepithelialization and improved healing outcomes. Studies have shown that EWDs can enhance wound closure rates compared to conventional treatments. Specifically, Haidari et al. (2020) even state that overaccumulation of silver into wounds has been proven to impair wound healing and that the usage of silver dressings can even increase “host tissue toxicity,” thus requiring careful attention when used [25].

When the skin is wounded, the disruption of the skin’s transepithelial potential (TEP) creates endogenous lateral electric fields at the wound site that are critical for the body’s natural reepithelialization mechanism. These lateral electric fields guide keratinocyte migration toward the wound edges to facilitate closure of the wound via galvanotaxis [26]. EWDs can replicate or enhance these natural electric fields by generating low-level electrical currents. These currents create a sustained and directed electric field, amplifying the natural bioelectric signal of the wound and providing a consistent stimulus for keratinocyte migration toward the wound center, leading to more efficient reepithelialization and wound healing [4].

Study limitations

Despite the promising results, it is important that we acknowledge the limitations of our study. With a population size of 100 patients, our study has a moderate sample size, thus limiting the generalizability of our findings. Our study did not include a control group who underwent sternal wound closure without the use of an EWD. This makes it difficult to definitively attribute the observed outcomes to the use of EWD alone. Consequently, a statistical comparison of procedure times with and without EWD also could not be made. Instead, the claims regarding the minimal effects of EWD usage on procedure times are supported by experiential observations rather than direct statistical analysis. All surgical procedures were performed by a single surgeon, which may limit the validity of our findings in other settings. While our 30-day follow-up provides valuable short-term data on wound healing, we are unable to assess the medium- to long-term improvement in wound healing through the use of the EWD. While we focused on key complications like infection and dehiscence, we did not assess other potential outcomes such as patient satisfaction or quality of life measures. Lastly, the nonblinded nature of the study could have introduced bias in the assessment of subjective outcomes like pain.

## Conclusions

Increasingly complex cardiac surgery patients (older, frailer, immunocompromised, obese) face high risks of sternal wound complications. This study provides an analysis of a complex patient population for an EWD for median sternotomy incisions. EWDs have been previously shown to disrupt biofilm formation as well as promote wound healing. We showed no sternal wound dehiscence or significant pain at the 30-day follow-up, with wound infection and significant pain at 14 days being rare, despite the high-risk nature of these patients. While promising for reducing postoperative complications, larger prospective studies with longer follow-up and comparison to sternal closure without EWD are needed to confirm the efficacy and safety of EWDs in cardiac surgery wound closure.
